# Apical constriction and epithelial invagination are regulated by BMP activity

**DOI:** 10.1242/bio.015263

**Published:** 2015-11-30

**Authors:** Vijay K. Jidigam, Raghuraman C. Srinivasan, Cedric Patthey, Lena Gunhaga

**Affiliations:** Umeå Centre for Molecular Medicine, Umeå University, Umeå S-901 87, Sweden

**Keywords:** BMP, F-actin, Invagination, RhoA, Apical constriction, Placodes

## Abstract

Epithelial invagination is a morphological process in which flat cell sheets transform into three-dimensional structures through bending of the tissue. It is accompanied by apical constriction, in which the apical cell surface is reduced in relation to the basal cell surface. Although much is known about the intra-cellular molecular machinery driving apical constriction and epithelial invagination, information of how extra-cellular signals affect these processes remains insufficient. In this study we have established several *in vivo* assays of placodal invagination to explore whether the external signal BMP regulates processes connected to epithelial invagination. By inhibiting BMP activity in prospective cranial placodes, we provide evidence that BMP signals are required for RhoA and F-actin rearrangements, apical constriction, cell elongation and epithelial invagination. The failure of placode invagination after BMP inhibition appears to be a direct consequence of disrupted apical accumulation of RhoA and F-actin, rather than changes in cell death or proliferation. In addition, our results show that epithelial invagination and acquisition of placode-specific identities are two distinct and separable developmental processes. In summary, our results provide evidence that BMP signals promote epithelial invagination by acting upstream of the intracellular molecular machinery that drives apical constriction and cell elongation.

## Abstract (precis)

**Summary:** We describe a novel role for BMP activity in promoting a direct and cell type-independent mechanism for apical constriction, cell elongation and epithelial invagination, separate from acquisition of placode-specific identities.

## INTRODUCTION

Epithelial invagination is a morphological process in which flat cell sheets transform into three-dimensional structures, like an epithelial pit/sac/cup or a furrow. The process of invagination is crucial during development as it plays an important role for the formation of the lens, inner ear, nasal cavity, adenohypophysis, trachea and salivary glands – all of which are different pit/sac/cup structures. Processes that involve furrow formation are gastrulation and neural tube bending, as well as the development of the *Drosophila* eye. Although many studies have addressed the intra-cellular molecular machinery involved during epithelial invagination (reviewed in Chauhan et al., 2015; Kondo and Hayashi, 2015; Martin and Goldstein, 2014; Sawyer et al., 2010), less is known about extra-cellular signals that affect this process.

Epithelial invagination is accompanied by apical constriction and cell elongation along their apical-basal axis (reviewed in Chauhan et al., 2015; Kondo and Hayashi, 2015; Martin and Goldstein, 2014; Sawyer et al., 2010). During apical constriction, the apical cell surface is reduced in relation to the basal cell surface, resulting in a cell shape change from cylindrical to conical or wedge shape. Apical constriction is considered to be driven by actin-myosin contraction, in which motor non-muscle Myosin II brings actin filaments together to generate contractile forces at the apical cell surface. Thus, apical constriction is mediated by the accumulation of F-actin at the apical side and appears to be a prerequisite for epithelial invagination of sensory placodes (Borges et al., 2011; Christophorou et al., 2010; Plageman et al., 2011, 2010; Sai and Ladher, 2008). Both *in vitro* and *in vivo* experiments have shown that blocking Myosin II or F-actin polymerization results in failure of placode invagination (Borges et al., 2011; Plageman et al., 2011; Sai and Ladher, 2008). Furthermore, apical localization of the Rho family GTPase RhoA has been shown to regulate the process of apical constriction, in part by regulating the accumulation of phosphorylated Myosin II (pMyosin) (Borges et al., 2011; Plageman et al., 2011). RhoA forms a complex together with Trio, Shroom3 and Rock1/2, and inhibition of either RhoA, Rock1/2 or Shroom3 activity disrupts apical constriction and placode invagination (Borges et al., 2011; Chauhan et al., 2011; Das et al., 2014; Lang et al., 2014; Plageman et al., 2011, 2010; Sai et al., 2014). However, how external signaling pathways regulate apical constriction and invagination has been poorly defined.

Cranial placodes found in chick and mouse are transient thickenings of the vertebrate embryonic head ectoderm that will give rise to sensory (olfactory, lens, trigeminal, otic and epibranchial) and non-sensory (hypophyseal) components of the peripheral nervous system (PNS) (reviewed in Baker and Bronner-Fraser, 2001; Jidigam and Gunhaga, 2013). Placodal cells derive from the neural plate border region, in which Bone Morphogenetic Protein (BMP) activity play an important role for its specification at late blastula stages (Linker et al., 2009; Patthey et al., 2009). Moreover, BMP signals are also required for the individual specification of placodal cell types at later stages (Brugmann et al., 2004; Glavic et al., 2004; Nguyen et al., 1998; Patthey et al., 2008; Sjödal et al., 2007). In most vertebrate embryos, three sensory placodes, the olfactory, lens and otic, and the non-sensory hypophyseal placode undergo invagination. Invagination is initiated soon after cell elongation and thickening of the epithelium, and involves inwards bending of the epithelium resulting in the formation of a cup-shape structure, often referred to as pit-structures. Whether the initial process of placode invagination is regulated by a common molecular mechanism, or is controlled by unique molecular codes for each individual placode remains to be determined.

In this study we have established *in vivo* assays of chick olfactory, lens, otic and hypophyseal placode invagination to explore how BMP activity regulates processes connected to epithelial invagination. Our *in vivo* results provide evidence that RhoA and F-actin rearrangements, apical constriction, cell elongation and epithelial invagination are regulated by a common BMP-dependent molecular mechanism. We conclude that the failure of placode invagination in BMP loss-of-function experiments is not caused by alterations in cell death or proliferation, or loss of placodal identity, but rather as a direct result of disrupted apical accumulation of RhoA and F-actin, which leads to lack of apical constriction and reduced cell length. In addition, our results show that epithelial invagination and acquisition of placode-specific identities are two independent developmental processes.

## RESULTS

### BMP activity is detected in placodal regions around the time of invagination

**Fig. 1.**
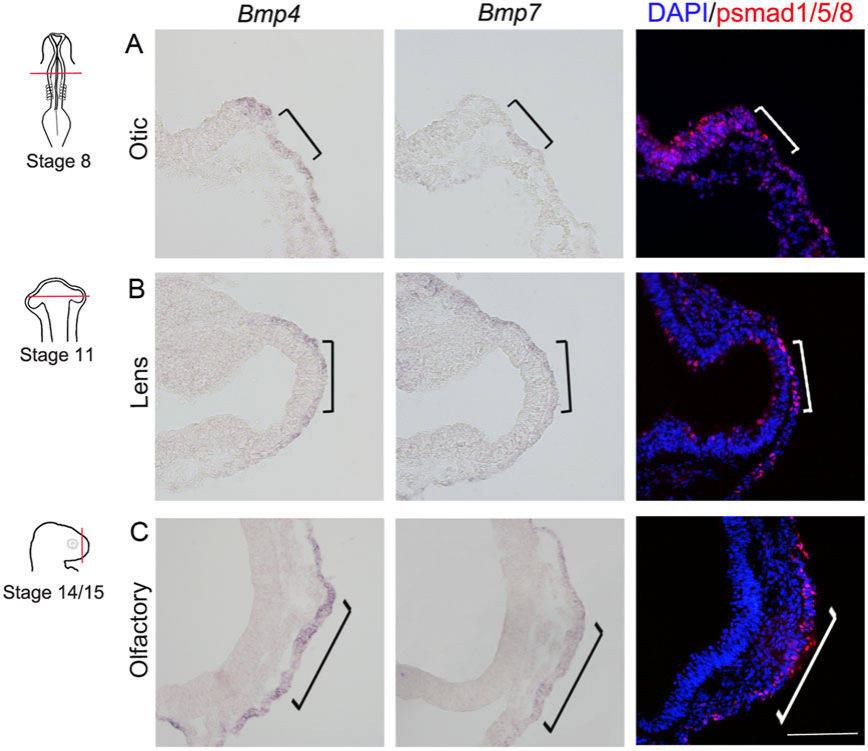
**BMP expression patterns in otic, lens and olfactory placodes.** (A) At stage 8, *Bmp4* and low levels of *Bmp7* expression, and immunoreactivity for p-Smad1/5/8 were detected in the otic placodal region (indicated by square brackets). (B) At stage 11, *Bmp4* and *Bmp7* expression, and immunoreactivity for p-Smad1/5/8 were detected in the lens placodal region (indicated by square brackets). (C) At stage 14, *Bmp4* and *Bmp7* expression, and immunoreactivity for p-Smad1/5/8 were detected in the olfactory placodal region (indicated by square brackets). Scale bar: 100 µm.

To study invagination we first characterized the timing of initial invagination in the olfactory, lens and otic sensory placodes in chick (*Gallus gallus*). Invagination begins around Hamburger and Hamilton (1951) stage 10/11 in the otic placode (Fig. S1A,B), stage 14 in the lens placode (Fig. S1C,D), and stage 17 in the olfactory placode (Fig. S1E,F). BMP signals have been shown to play an important role for the generation of placodes (Brugmann et al., 2004; Glavic et al., 2004; Linker et al., 2009; Nguyen et al., 1998; Patthey et al., 2009, 2008; Sjödal et al., 2007). To address whether BMP activity might play a role in the invagination of placodes, we first examined the expression pattern of *Bmp4* and *Bmp7*, as well as the location of its downstream signaling mediator phosphorylated (p)Smad1/5/8 prior to and around the time of placode invagination. At stages 8-10, *Bmp4* and *Bmp7* expression and pSmad1/5/8 are detected in the otic placode region (Fig. 1A; data not shown). Moreover, at stages 11-14, and stage 14-17, *Bmp4* and *Bmp7* expression and pSmad1/5/8 are observed in the lens and olfactory placodal domains, respectively (Fig. 1B,C, data not shown). Thus, in the olfactory, lens and otic placodal regions *Bmp4*, *Bmp7* expression and pSmad1/5/8, indicative of BMP activity, are detected at the time point for initiation of sensory placode formation and invagination.

### BMP signals are required for placode invagination

To examine whether BMP signals are required for the invagination of sensory placodes, we performed *in vivo* experiments in which BMP activity was inhibited. This was accomplished by *in ovo* electroporation of a construct expressing the BMP antagonist Noggin in olfactory and lens placodal regions. A green fluorescent protein (GFP) vector was transferred alone or together with the Noggin-expressing vector in the ectodermal region of interest prior to the onset of invagination. After culture, successfully electroporated embryos with GFP staining within the lens or olfactory regions were analyzed.

**Fig. 2.**
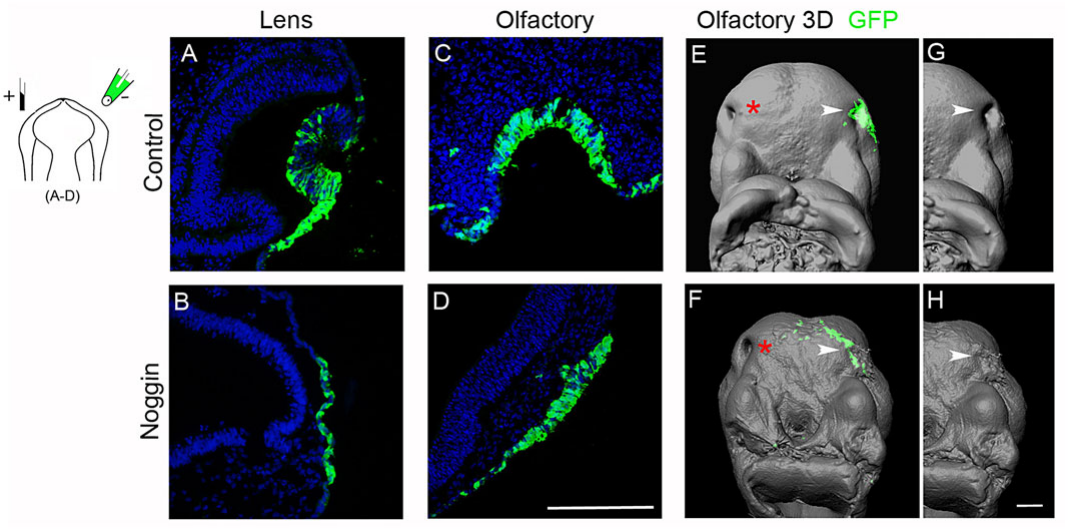
**BMP activity is required for lens and olfactory epithelial invagination.** Stage 11 chick electroporated with GFP (green) alone (A,C,E,G) or together with Noggin (B,D,F,H) in lens or olfactory placodal regions, and cultured to approximately stage 15 and 20, respectively. (A-D) Nuclei are detected with DAPI. (A,C) Control embryos electroporated with GFP in lens and olfactory gives rise to normal placode invagination. (B) Lens ectoderm co-electroporated with a Noggin-construct failed to develop a placode and to invaginate. (D) Olfactory epithelium co-electroporated with a Noggin-construct developed a small placode, but failed to invaginate. (E-H) 3D OPT images visualizing lack of the olfactory pit after BMP inhibition. White arrow heads indicate the electroporated olfactory regions, and red asterisks indicate the contralateral non-electroporated olfactory epithelium. Scale bars: 100 µm in A-D, 400 µm in E-H.

All control lens electroporated embryos exhibited normal morphology of the lens epithelium (Fig. 2A; *n*=12/12). In contrast, when Noggin was electroporated in the lens, the invagination was suppressed and no thickened placode formed (Fig. 2B; *n*=15/15). Also control olfactory electroporated embryos exhibited normal morphology of the olfactory epithelium (Fig. 2C; *n*=21/21), whereas all Noggin-electroporated embryos exhibited disturbed invagination of the olfactory epithelium (Fig. 2D; *n*=21/21). On occasion, and depending on the electroporation efficiency, a small invagination in a GFP negative region of the electroporated olfactory placodal domain could be detected (Fig. S2B). The majority of the Noggin-electroporated olfactory regions still possessed a placode (*n*=18/21), as observed by the thickened ectoderm (Fig. 2D, *n*=12/21; Fig. S2A, *n*=5/21; Fig. S2B, *n*=1/21), while a few embryos completely lacked placode formation in the Noggin-electroporated side (Fig. S2C; *n*=3/21). Using a whole embryo three-dimensional (3D)-OPT imaging technique, the disrupted invagination of the olfactory placode and lack of pit formation was clearly visible after BMP inhibition (*n*=4/4), compared to control embryos (*n*=4/4) (Fig. 2E-H; Movies 1, 2).

**Fig. 3.**
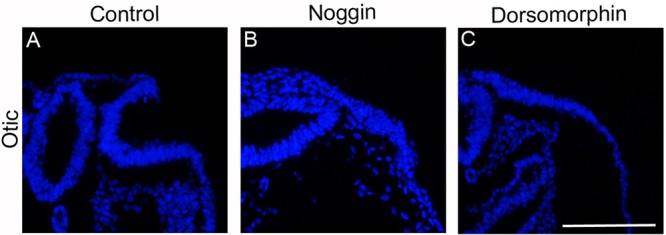
**Otic placode fail to invaginate in the absence of BMP.** (A) *Ex ovo* cultures of stage 7 embryos cultured to stage 12 exhibited normal otic placode invagination. (B,C) In the presence of Noggin (B) or Dorsomorphin (C), the otic placode developed, but failed to invaginate. Nuclei are detected with DAPI. Scale bar: 100 µm.

We next analyzed the invagination process of the otic placode. Electroporating stage 6/7 embryos in the otic region, which lies in the proximity of the heart region, consistently resulted in heart malformation and arrested development (data not shown), preventing analysis of otic placode invagination. To circumvent this issue, we used a modified Cornish Pasty method (Nagai et al., 2011), which is another type of *ex ovo*, whole embryo culture technique. Stage 6/7 embryos were cultured in control medium alone or together with Noggin or Dorsomorphin, a specific BMP receptor inhibitor, to approximately stage 12/13. In all control embryos, the otic placode underwent invagination (Fig. 3A; *n*=13/13), whereas the otic placode in BMP-inhibited embryos failed to invaginate (Fig. 3B,C; *n*=17/17). The majority of the BMP-inhibited embryos still possessed an otic placode (Fig. 3B,C; *n*=15/17), and only a few embryos completely lacked otic placode formation (Fig. S2D; *n*=2/17). Taken together, the process of cell elongation in the olfactory and otic placodes appears to be BMP independent, whereas the invagination process of olfactory, lens and otic ectoderm all require BMP activity. These data suggest that placode formation and invagination are two independent processes, and that BMP activity regulates a general mechanism for invagination of sensory placodes.

### Loss of invagination after BMP inhibition is not due to loss of placode identity

At earlier stages, BMP activity has been shown to be important for the specification of placodal progenitors (Brugmann et al., 2004; Glavic et al., 2004; Nguyen et al., 1998; Patthey et al., 2008; Sjödal et al., 2007). Moreover, previous studies have indicated that BMP signals are required for the specification of lens fibre cells from stage 4 until stage 13, in part by regulating the onset of L-Maf (Pandit et al., 2011; Sjödal et al., 2007), which also was verified in this study (Fig. S3). Therefore, we examined if the disrupted invagination in the BMP loss-of-function experiments might be a consequence of disturbed acquisition of placodal cell identity in the olfactory and otic placodes, or whether at this stage BMP activity is required specifically for the invagination process. To address this issue, we examined whether differentiated cell types were generated in placodes of Noggin-treated embryos, using a set of molecular markers that uniquely define specific placodes.

**Fig. 4.**
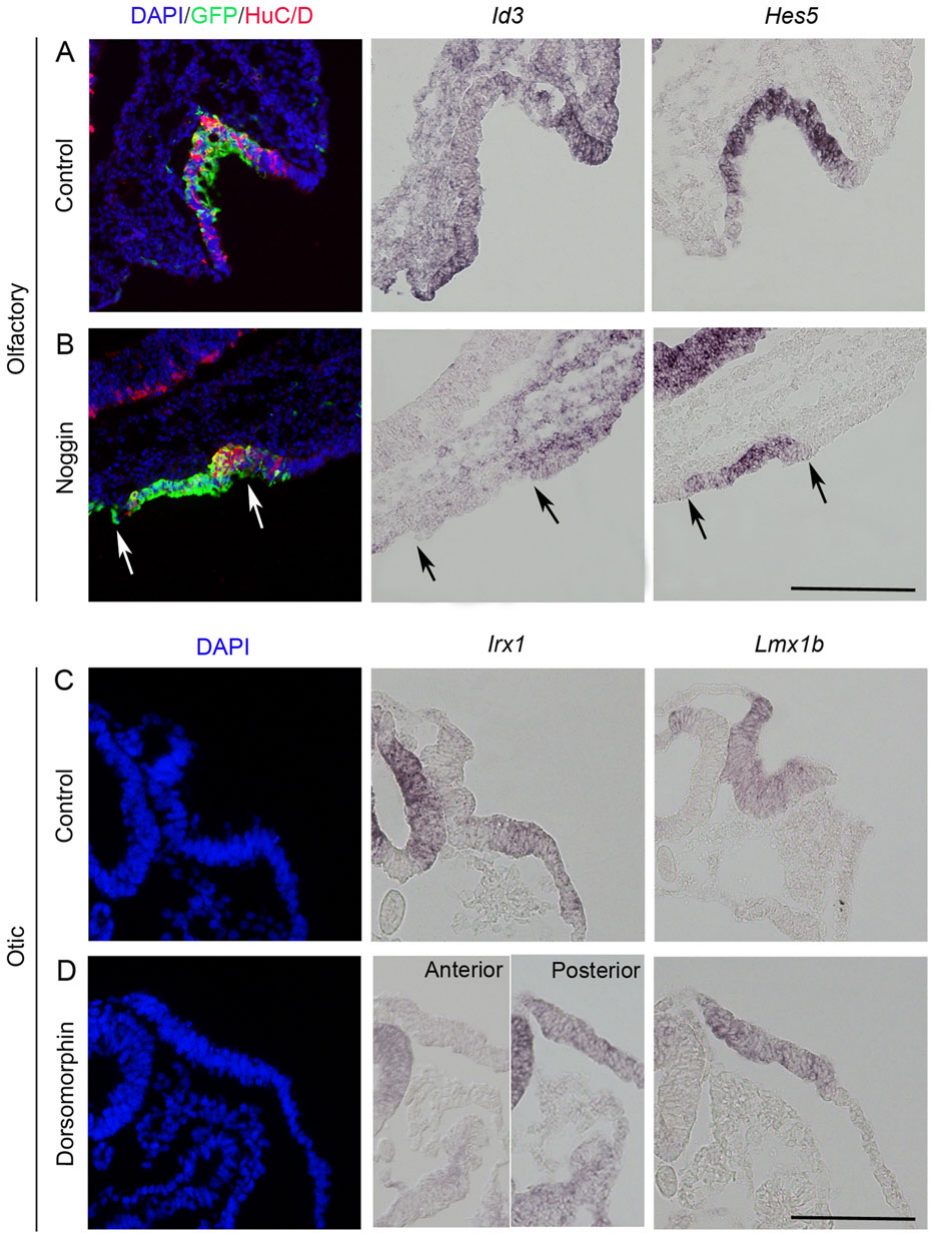
**Placodal cell identity is maintained in the olfactory and otic epithelium after BMP inhibition.** (A,B) *In ovo* electroporation of stage 11/12 embryos in the olfactory placodal region using GFP alone (A, *n*=3) or together with Noggin (B, *n*=4) and cultured to stage 19/20. (A) Control-electroporated embryos show HuC/D and *Hes5* expression observed throughout the sensory part of the olfactory epithelium and *Id3* in the respiratory part of the epithelium (*n*=3/3). (B) Noggin electroporation resulted in reduced or loss of *Id3* expression. *Hes5* expression and reduced expression of HuC/D was detected in the flat olfactory placodal region (*n*=4/4). Arrows indicate the electroporated region. (C,D) Stage 7 chick embryos cultured to stage 12 alone (C, *n*=3) or in the presence of Dorsomorphin (D, *n*=3). Both control (C, *n*=3/3) and Dorsomorphin-treated (D, *n*=3/3) embryos expressed *Irx1* and *Lmx1b* in the otic epithelium. Scale bars: 100 µm.

The olfactory placode normally give rise to sensory and respiratory olfactory cells (Maier et al., 2010). The sensory domain includes stem-cell like *Hes5*^+^ cells and post-mitotic HuC/D^+^ neurons, whereas cells in the respiratory region express *Id3* (Maier et al., 2010). In addition, *Id3* is a direct target gene of BMP signaling, and can be used as readout of BMP activity (Hollnagel et al., 1999). All control embryos electroporated in the olfactory placodal region expressed *Hes5* and HuC/D in the sensory domain and *Id3* in the respiratory region of the invaginated olfactory pit (Fig. 4A; *n*=3/3). In contrast, inhibition of BMP suppressed the generation of *Id3*^+^ respiratory cells, (Fig. 4B; *n*=4/4) which confirmed the suppression of BMP activity and indicated a loss of respiratory cell types in line with previous results (Maier et al., 2010). However, *Hes5* and HuC/D expression were still detected in the flat olfactory epithelium (Fig. 4B; *n*=4/4). Thus, although BMP signals are required for the specification of olfactory respiratory cells at this stage, the generation of placodal cells with an olfactory sensory identity are not dependent on BMP signals.

In the otic placode, *Irx1* and *Lmx1b* are markers of the posterior non-neurogenic otic epithelium, and consistently control electroporated embryos expressed these markers in the otic placode (Fig. 4C; *n*=3/3). In the BMP-inhibited flat otic epithelium, *Lmx1b*^+^ cells were generated throughout the otic region, whereas *Irx1*^+^ cells were detected in the posterior part of the otic region (Fig. 4D; *n*=3/3). These results indicate that at this stage the specification of otic placodal cells occurs independently of BMP activity. Together, these results provide evidence that cells can differentiate beyond the placode stage and mature into differentiated cell types in the absence of invagination. Thus, the acquisition of placode-specific cell types and placode invagination are independent processes.

### Disturbed invagination after BMP inhibition is not due to changes in cell death or proliferation

We next analyzed whether inhibition of BMP activity caused a reduction of the proliferation rate and/or increased cell death in the prospective placodal epithelium, thereby leading to disrupted invagination of the sensory placodes. For this purpose we examined the expression of the cell proliferation marker phosphorylated HistoneH3 (pHH3) and the cell death marker activated (a) Caspase3 in control and BMP-inhibited embryos (either Noggin-electroporated in the lens and olfactory regions, or Dorsomorphin treated embryos for the otic region). Quantification of aCaspase3^+^/GFP^+^ double-positive cells revealed no significant changes in aCaspase3^+^ cells in the Noggin-electroporated lens or olfactory ectodermal region compared to control embryos (Fig. S4A,B). Moreover, no significant change in aCaspase3^+^ cells in the otic ectodermal region in Dorsomorphin-treated embryos was observed compared to control embryos (Fig. S4C). Regarding proliferation, there was no significant change in pHH3^+^/GFP^+^ double-positive cells in the Noggin-electroporated lens (Fig. S4D), and no significant change in pHH3^+^ cells in the otic region of Dorsomorphin-treated embryos compared to control embryos (Fig. S4F). On the other hand a significant decrease in pHH3^+^ cells was observed in the Noggin-electroporated olfactory region compared to control embryos (Fig. S4E). These data suggest that, although reduced cell proliferation in the olfactory placode might affect the invagination process, reduced cell proliferation or increased apoptosis in general cannot explain failure in placode invagination after BMP inhibition.

### BMP inhibition prevents cell shape changes in prospective placodal cells

**Fig. 5.**
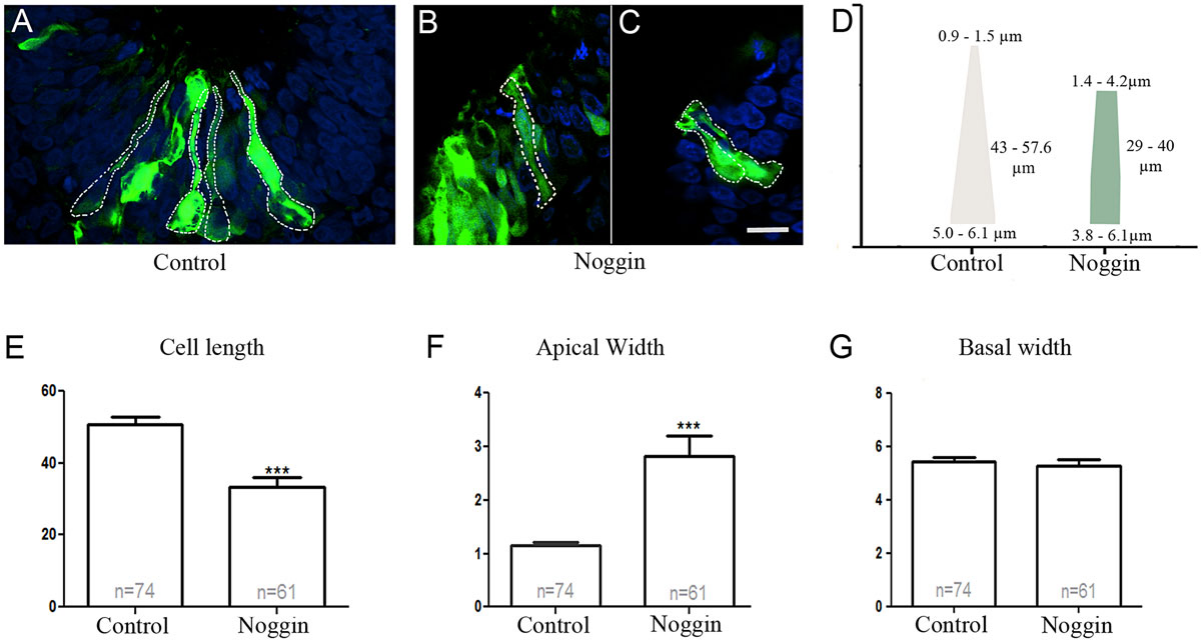
**Decreased apical width and cell elongation after BMP inhibition in the olfactory placode.** (A-C) *In ovo* electroporation of stage 11/12 embryos in the olfactory placode with GFP alone (A) or Noggin (B,C), and cultured to approximately stage 20. The cell shape of GFP^+^ cells was determined on 30 µm cryosections (dashed lines) of PFA-fixed tissue. The images were processed to measure cell length as well as apical and basal cell width. Scale bar: 10 µm. (D) Model image with the span of cell dimensions depicted in µm for control and Noggin-electroporated cells. (E-G) The average apical and basal width of cells, and cell length was quantified and depicted in the graphs. (E) The cell length was significantly shorter in Noggin-electroporated cells (*P*=0.000787). (F) The apical width was significantly wider in Noggin-electroporated cells (*P*=0.0008). (G) The basal width was not significantly altered in Noggin-electroporated cells compared to control cells (*P*=0.6033). Error bars represent s.e.m., ***P<0.0001.

Cell elongation and apical constriction facilitates the invagination process. To analyze whether BMP inhibition disrupted cell elongation and/or apical constriction, we measured the cell length, basal cell width and the apical cell width in control and BMP-inhibited placode regions. The olfactory placode was the optimal tissue to use for these studies, since BMP-inhibition resulted in a lack of invagination, but did not affect placode formation, and GFP-electroporated cells could be readily visualized to examine cell size and shape (Fig. 2D). GFP^+^ cells from the medial invaginated part of the olfactory pit were analyzed in both control (*n*=8) and Noggin treated (*n*=8) embryos, with a total of 74 control cells and 61 Noggin-treated cells (Fig. 5).

BMP inhibition in the olfactory region significantly reduced the cell length from an average of 50 µm in the control to an average of 33 µm in the Noggin-electroporated cells (Fig. 5A-E). In addition, the apical width of the Noggin-electroporated cells was significantly increased (average of ∼2.8 µm) compared to control cells (average of ∼1.2 µm) (Fig. 5D,F), whereas the average basal width was not significantly changed (Noggin ∼5 µm vs control ∼5.5 µm) (Fig. 5D,G). This resulted in an apical/basal ratio of 0.56 for Noggin-electroporated cells compared to 0.22 for control embryos. Thus, in placodal cells deprived of BMP activity cell elongation was reduced and apical constriction was abrogated.

### BMP inhibition disrupts apical accumulation of RhoA and F-actin

**Fig. 6.**
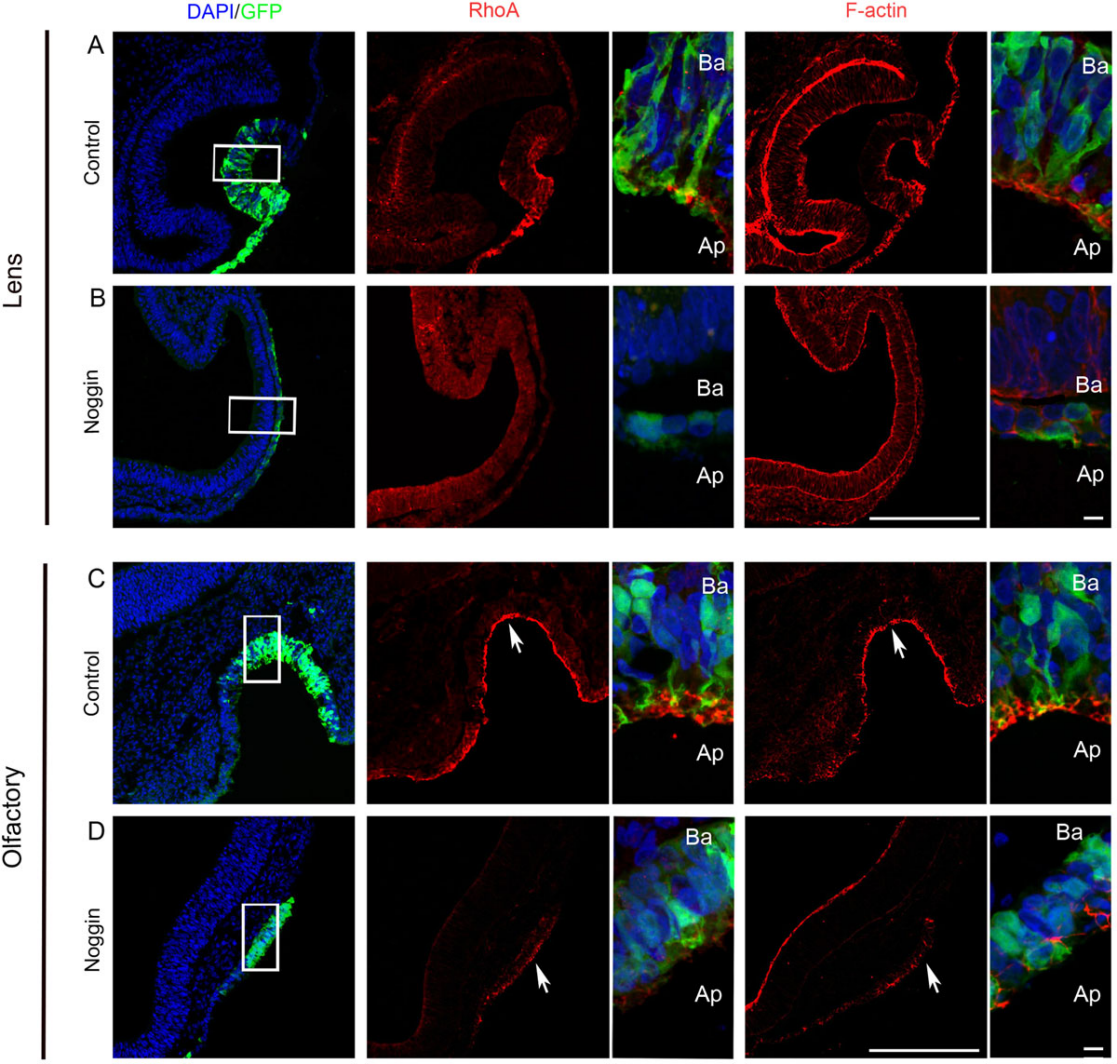
**Disrupted apical accumulation of RhoA and F-actin after BMP inhibition.** (A-D) *In ovo* electroporation of stage 10/11 embryos in the prospective lens and olfactory ectoderm region using a GFP reporter construct alone (A, *n*=5; C, *n*=6) or together with a Noggin construct (B, *n*=7; D, *n*=7) and cultured to stage 15/16 (lens) and stage 19/20 (olfactory). (A,C) All control embryos show accumulated expression of RhoA and F-actin at the apical region of the cells (A, *n*=5/5; C, *n*=6/6). (B,D) Inhibition of BMP signaling disturbed the apical accumulation of RhoA and F-actin in both lens and olfactory epithelial cells (B, *n*=7/7; D, *n*=7/7). Boxed regions indicate the areas of higher magnification images. Arrows in C and D indicate the apical side. Scale bars: 100 µm (lower magnification); 10 µm (higher magnification). Ap, apical; Ba, basal.

**Fig. 7.**
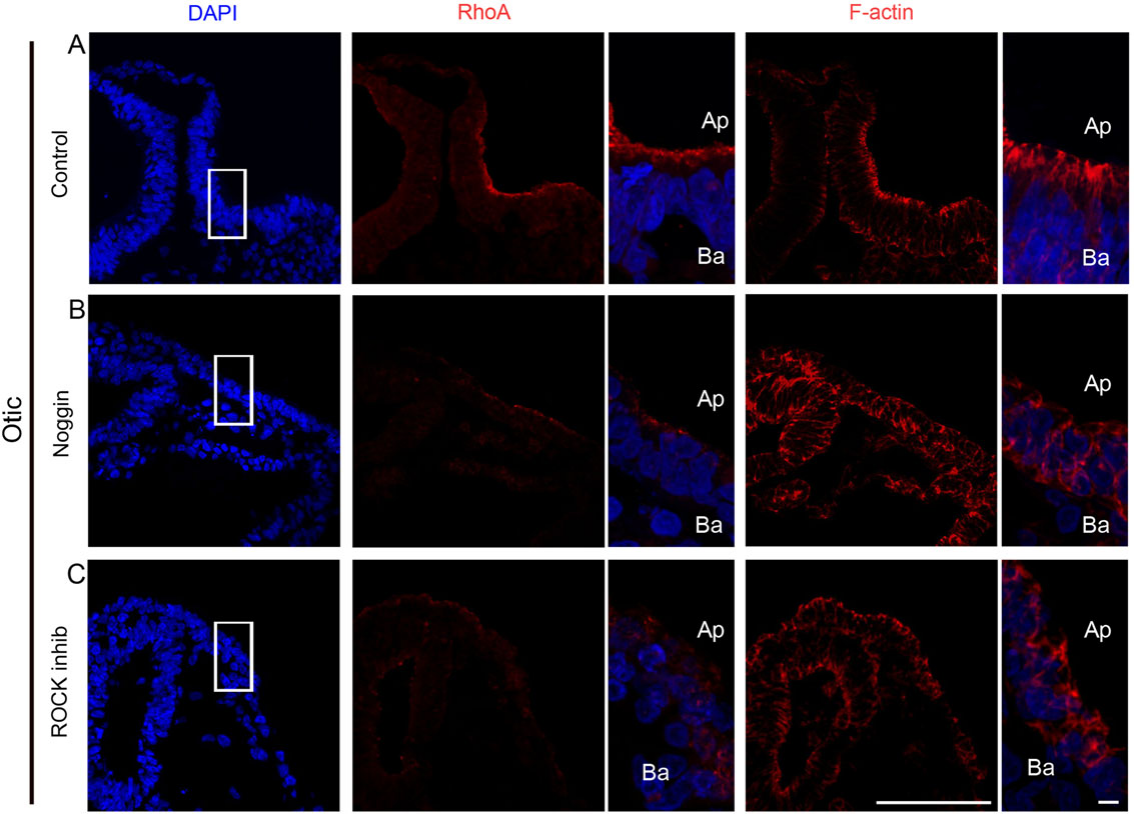
**Disrupted apical accumulation of RhoA and F-actin in the otic placode after BMP or ROCK inhibition.** (A-C) *Ex-ovo* cultures of whole stage 6/7 embryos alone (A, *n*=5) or together with Noggin (B, *n*=7) or the ROCK inhibitor Y27632 (C, *n*=4), and cultured to approximately stage 12. (A) In control embryos, RhoA was apically localized with apical F-actin polarization (*n*=5/5). (B) Exposure to Noggin prevented RhoA and F-actin accumulation (*n*=7/7). (C) Embryos treated with the ROCK inhibitor exhibited disturbed apical accumulation of RhoA and F-actin (*n*=4/4). Boxed regions indicate the areas of higher magnification images. Scale bar: 100 µm (lower magnification); 10 µm (higher magnifications). Ap, apical; Ba, basal.

Apical constriction and placode invagination have been shown to be mediated by apical accumulation of RhoA that facilitate apical localization of F-actin, which in turns activates the actin-myosin network (Plageman et al., 2011, 2010; Sai and Ladher, 2008). Using confocal microscopy, we analyzed whether the accumulation of RhoA and the apical restriction of F-actin were disturbed in the BMP inhibited placodal regions. In all three invaginating sensory placodes, the normal apical localization of RhoA and F-actin was lost upon BMP-inhibition (Fig. 6A-D; Fig. 7A,B). In the BMP inhibited embryos, the expression of RhoA was severely reduced or completely lost, and the cellular sub-localization of F-actin, visualized by Phalloidin staining, was changed to the periphery of the ectodermal cells (Fig. 6B,D; Fig. 7B) instead of being apically restricted as seen in control embryos (Fig. 6A,C; Fig. 7A). These results suggest that BMP activity is required for proper accumulation of RhoA and F-actin at the apical side of the cells and for subsequent apical constriction.

To further assess a general requirement for BMP signals during epithelial invagination we also analysed the hypophyseal non-sensory placode, by electroporating the Noggin construct in the hypophyseal region at stage 12 and cultured to approximately stage 15/16. Control embryos showed accumulated expression of F-actin at the apical region of the cells, and an invagination of the hypophyseal epithelium (Fig. S5A; *n*=4). Inhibition of BMP activity disrupted the apical accumulation of F-actin, and suppressed the invagination of the hypophyseal epithelium (Fig. S5B; *n*=5). RhoA was not expressed at the examined stages in the hypophyseal epithelium (Fig. S5; *n*=5). Thus, BMP signals are required for F-actin accumulation and the invagination process in both sensory and non-sensory placodes.

### Blocking ROCK activity mimics BMP inhibition and disrupts placode invagination

RhoA is known to form a complex together with Trio, Shroom3 and Rock1/2, and inhibition of either RhoA or Rock1/2 activity disrupts apical constriction and placode invagination (Borges et al., 2011; Plageman et al., 2011; Sai et al., 2014). To directly evaluate and compare our BMP-inhibition results with experiments inhibiting ROCK activity, we used the Cornish pasty method to culture whole embryos together with Y27632, a pharmacological ROCK inhibitor. During these conditions the otic, lens and olfactory placodes failed to invaginate (Fig. 7C; Fig. S6B,D), in a similar manner as after BMP inhibition (Fig. 7B; Fig. 6B,D). In addition, confocal microscopy showed that the expression of RhoA was severely reduced or completely lost, and F-actin was located at the periphery of the ectodermal cells instead of being apically restricted as seen in control embryos (Fig. 7A,C; Fig. S6A-D). Notably, lens placode formation was visible after ROCK inhibition (Fig. S6B, *n*=5/5), and under these conditions, *L-Maf*^+^ and δ-crystallin^+^ lens fiber cells were still generated (Fig. S3C,D). Thus, blocking ROCK activity, results in failure in apical constriction and loss of invagination, phenotypes that are similar to or indistinguishable from BMP-inhibited sensory placodes. Together these results indicate that BMP signals act upstream of ROCK activity during apical constriction and epithelial invagination.

## DISCUSSION

### BMP is required for epithelial invagination

By studying three different sensory placodes and one non-sensory placode we have unraveled a common mechanism, in which BMP activity is required for epithelial invagination. Our data in chick show that expression of BMP ligands and pSmad1/5/8 is detected in the placodal regions prior to and around the onset of epithelial invagination. Consistently, in mouse, targeted deletions of different components of the BMP pathway lead to disturbed lens vesicle and olfactory pit formation (Beebe et al., 2004; Furuta and Hogan, 1998; Maier et al., 2010; Wawersik et al., 1999). In addition, our previous results in chick have indicated that defective BMP signaling disrupts lens and olfactory placode invagination (Maier et al., 2010; Pandit et al., 2011). Our present results from studying the lens, olfactory, otic and hypophyseal placodes suggest that neither loss of placodal identity, reduced cell proliferation or increased cell death is causing the disturbed placode invagination after BMP inhibition. In agreement with this, it has previously been shown that disturbed invagination of the mouse otic placode, caused by Gata3 deficiency, is not due to increased apoptosis (Lilleväli et al., 2006). In addition, our results in the olfactory and otic placodes indicate that the process of cell elongation, which enables thickening of the ectoderm, is BMP independent. Subsequently, a disturbed placode invagination following attenuation of BMP signaling is not caused by a failure in placode formation.

Though BMP activity plays an important role for epithelial invagination, it is not the only signaling pathway that regulates this complex process. Studies in chick and mouse have shown that FGF activity is required for proper invagination of the olfactory and otic placodes (Maier et al., 2010; Sai and Ladher, 2008; Zelarayan et al., 2007). In addition, using chick otic tissue cultures it has been suggested that FGF activity plays a critical role for the activation of myosin II and reorganization of F-actin, followed by placode invagination (Sai and Ladher, 2008). Knockdown of FGF3 expression using silencing (si) RNA in the developing otic region in chick leads to partially invaginated placodes or failure in otic vesicle formation (Zelarayan et al., 2007). Moreover, Gata3 deficient mouse embryos exhibit a loss of *Fgf10* expression in the otic epithelium followed by disrupted otic placode invagination (Lilleväli et al., 2006), and FGF signaling has been shown to be required for apical constriction in the zebrafish lateral line primordium (Harding and Nechiporuk, 2012). In the ascidian tunicate *Ciona*, inhibition of the FGF signaling pathway results in disrupted invagination of the atrial ectoderm and disrupted formation of the atrial siphon (Kourakis and Smith, 2007), a structure that has been proposed homologous to otic placode derivatives (Mazet and Shimeld, 2005; Wada et al., 1998). Taken together, these findings indicate that both BMP and FGF signals play important roles to control invagination. Thorough studies remain to be performed to reveal whether balanced activity of FGF and BMP signals regulate the down-stream mechanism that regulates the invagination process.

### BMP activity acts up-stream of RhoA and actin-myosin contractility

Our results provide evidence that loss of BMP activity in prospective placodal regions of the head ectoderm disrupts accumulation of RhoA and F-actin at the apical side of the placodal cells and for subsequent apical constriction. In support of this, Beebe and colleagues have shown that lens ectodermal cells, in which the type I receptors Bmpr1 and Acvr1 are deleted, failed to reorganize F-actin to their apical side and the lens epithelium did not invaginate properly (Rajagopal et al., 2009). Moreover, in *Drosophila*, the orthologue of BMP2/4, Dpp, promotes apical constriction in the eye imaginal disc via regulation of integrin expression and stabilization of microtubules (Fernandes et al., 2014). Several studies have shown that apical constriction of F-actin is required to activate myosin in the apical cortex of these cells, and subsequently for functional actin–myosin contractility (reviewed in Martin and Goldstein, 2014). In addition, many studies have contributed to findings how a complex of RhoA, Rock1/2, Trio and Shroom3 connects to F-actin and myosin to regulate apical constriction (reviewed in Chauhan et al., 2015; Kondo and Hayashi, 2015; Martin and Goldstein, 2014; Sawyer et al., 2010). Consistently, our results show that *in vivo* inhibition of ROCK activity disrupts RhoA and F-actin accumulation and the subsequent invagination process of sensory placodes. Moreover, our data provide evidence that morphologically the ROCK-depleted placodal epithelia were indistinguishable from the BMP-inhibited flat placodal domain, indicating that BMP activity acts up-stream of the ROCK-regulatory molecular machinery to regulate epithelial invagination.

Our results also show that suppressed BMP activity in placodal cells diminishes cell elongation. Consistently, the onset of Decapentaplegic (Dpp) signaling, the fly homolog of the vertebrate BMP signaling molecules, correlates with the cell elongation process in the developing *Drosophila* wing discs (Shen and Dahmann, 2005; Widmann and Dahmann, 2009). Moreover, Dahmann and colleagues have shown that Dpp signaling is both required and sufficient to regulate an elongated columnar cell shape by controlling the sub-cellular localization of activated Rho1 and Myosin II (Widmann and Dahmann, 2009). Interestingly, *Drosophila* mutations in *wollknauel* resulted in reduced Dpp signaling and impaired mesoderm invagination (Haecker et al., 2008). The existence of a general mechanism whereby BMP activity regulates morphogenesis is further supported by results in cell lines, in which both BMP2 and BMP7 induces actin reorganization in pluripotent C2C12 cells and fibroblast, respectively (Gamell et al., 2008; Konstantinidis et al., 2011). Taken together, BMP signals appear to activate a common regulatory pathway for actin rearrangement connected to cell and tissue morphogenesis, including epithelial invagination.

### Invagination and acquisition of placodal cell identity are independent developmental processes

BMP signals are known to be required for the specification of placodal cell types at early stages (Linker et al., 2009; Patthey et al., 2009). Our results now show that lack of invagination in BMP loss-of-function experiments is not a mere consequence of the loss of placodal cell identity, as shown by the fact that the flat otic and olfactory epithelia are still thickened, and cells within it still express individual placode-specific markers. Thus, at these stages, any changes in gene regulatory networks caused by the loss of BMP activity clearly do not act at the level of the placode versus non-placode cell type decision. This indicates that acquisition of placodal cell identity and epithelial invagination are two independent developmental processes that can be experimentally separated. In agreement with this, it has been shown that failure in epithelial apicobasal polarity and F-actin rearrangements during neural tube closure does not affect ventral or dorsal cell specification or cell differentiation (Eom et al., 2011, 2012). However, in the lens, our present and previous results provide evidence that BMP signals are required for the specification of lens fiber cells from stage 4 until stage 13, in part by regulating the onset of L-Maf (Pandit et al., 2011; Sjödal et al., 2007). Thus, in the lens, the two BMP-dependent processes of lens cell specification and invagination overlap in time, explaining why both lens identity and invagination are lost upon BMP inhibition. On the other hand, our data show that in ROCK loss-of-function experiments the lens ectoderm fails to invaginate, but still forms an *L-Maf*-positive placode, supporting our findings that acquisition of placodal cell identity and epithelial invagination can be separated. Whether BMP signaling promotes expression of effector genes related to apical constriction and morphogenetic movements, or whether its effects are mediated by post-translational modifications resulting into cytoskeletal re-arrangements remains to be elucidated.

In summary, we have established *in vivo* assays of epithelial invagination in chick enabling morphological and genetic observations of the invagination process in four different placodes. Our results provide evidence that BMP signals promote epithelial invagination by acting upstream of the intracellular molecular machinery that drives apical accumulation of RhoA and F-actin, and thereby apical constriction. Moreover, our results show that epithelial invagination and acquisition of placode-specific identities are two separable developmental processes. Taken together, our results suggest a novel role for BMP activity in promoting a specific and cell type-independent mechanism for apical constriction, cell elongation and epithelial invagination.

## MATERIALS AND METHODS

### Embryos

Fertilized white Leghorn chicken eggs were obtained from Strömbäcks Ägg, Umeå, Sweden. Chick (*Gallus gallus*) embryos were staged according to the protocol of Hamburger and Hamilton (1951).

### *In ovo* electroporation

Chick embryos were electroporated in the lens (stage 10/11), olfactory (stage 11-13) and hypophyseal (stage 12) ectoderm. Vectors used for electroporation were: pCAβ-*EGFP*-m5 (0.6-1 μg/μl) (Yaneza et al., 2002), pMiwIII –Noggin (0.61 μg/μl) (Timmer et al., 2002). Inhibition of BMP signaling by the Noggin-construct has previously been verified (Maier et al., 2010; Pandit et al., 2011). The DNA-constructs were transferred using an Electro Square Porator ECM 830 (BTX) by applying 5 pulses (9-15 V, 25 ms duration) at 1-s intervals. Embryos were further cultured *in ovo* to stage 15/16 for hypophyseal and lens studies, and stage 19/20 for olfactory placodal studies. Noggin/GFP-electroporated regions were compared with control GFP-electroporations.

### *Ex ovo* culture of the chick embryo

The modified Cornish pasty method (Nagai et al., 2011), an *in vivo*/*ex ovo* chick embryo culture technique, was used with slight changes. Briefly, stage 5-7 embryos were dissected and folded in half along the midline into a semi-circle, and cut along the outside edge. The embryos were allowed to rest undisturbed in Ringer's solution at room temperature (RT) for approximately 45-60 min. Embryos were then transferred to a 4-well plate containing 500 µl of medium (a 2:1 mixture of thin albumen and Ringer's solution with penicillin and streptomycin 5000 U/ml) alone or together with inhibitors. Each well contained one embryo. The cultures were grown at 37°C to approximately stage 9/10 for apoptosis and proliferation analyses in the otic placode, and to approximately stage 12/13 for all other otic studies. For olfactory and lens studies, embryo cultures were grown to stage 11, after which the embryos were exposed to Y27632 until the end of culture; stage 15/16 (lens) and stage 18 (olfactory). Inhibitors used were; Noggin condition medium, the BMP-receptor inhibitor Dorsomorphin (50 µM) (Stemgent), and the ROCK inhibitor Y27632 (40 µM) (Abcam). Soluble Noggin conditioned medium were obtained from stably transfected Chinese hamster ovary (CHO) cells (Lamb et al., 1993) and cultured in CHO-S-SFM II media (Gibco). Noggin conditioned media was used at an estimated concentration of 50 ng/ml. Inhibition of BMP signaling by the use of Noggin conditioned medium has previously been verified (Maier et al., 2010; Patthey et al., 2009).

### 3D analysis by optical projection tomography

Tissue preparation of chick heads for OPT scanning was essentially performed as previously described (Alanentalo et al., 2008). OPT scanning, using the Bioptonics 3001 OPT scanner (Bioptonics) visualizing anti-GFP-antibodies using goat anti-rabbit Alexa Fluor 488 (1:400) (Molecular Probes) was performed as previously described (Alanentalo et al., 2008).

### *In situ* hybridization and immunohistochemistry

*In situ* RNA hybridization and immunohistochemistry was performed essentially as described (Wilkinson and Nieto, 1993; Wittmann et al., 2014) on consecutive sections. Embryos were first fixed in 4% paraformaldehyde (PFA) for 1-2 h at 4°C, cryoprotected in 30% sucrose, embedded and cryosectioned at 10 µm. *In situ* hybridization was performed using the following Dig-labelled chick probes; *Bmp4* (Francis et al., 1994), *Bmp7* (Liem et al., 1995), *Hes5* (Fior and Henrique, 2005), *Id3* (Kee and Bronner-Fraser, 2001), *Irx1* (Abello et al., 2010), *L-Maf* (Reza and Yasuda, 2004), *Lmx1b* (Abello et al., 2010). Antibodies used were; anti-pHistoneH3 (Millipore, 1:500), anti-RhoA (Santa Cruz, 1:200) and anti-pSmad1/5/8 (Cell Signaling, 1:1000) rabbit antibodies, and anti-aCaspase3 (Cell Signaling, 1:1000) and anti-HuC/D (Molecular Probes, 1:200) mouse monoclonal antibodies, and anti-sheep δ-crystallin (Beebe and Piatigorsky, 1981; 1:1000). Secondary antibodies used were goat anti-rabbit Cy3 (1:400) (Molecular Probes), goat anti-mouse Alexa Fluor 594 (1:400) (Molecular Probes) and goat anti-sheep Alexa Fluor 594 (1:400) (Molecular Probes). Rhodamine Phalloidin were used to visualize F-actin (Molecular Probes), and nuclei were stained using DAPI (Sigma). Slides were mounted with Fluorescent or Glycergel mounting medium (Dako). Images were taken using a 20×/0.50 numerical aperture lens on a Nikon Eclipse E800 microscope, equipped with a CCD camera connected to a PC (Nikon Imaging Software NIS-Elements) at room temperature. Overly images were processed with Photoshop CS5 software (Adobe).

### Morphometric analysis

Stage 11/12 embryos were electroporated in the olfactory with GFP alone or together with Noggin and cultured until stage 19/20. Whole embryos were fixed in 4% PFA for 2 h, treated in 30% sucrose, embedded and cryosectioned at 30 µm. GFP^+^ cells from the medial invaginated part of the olfactory pit were analyzed using a 20×/0.50 and 60×/1.40 numerical aperture lenses on a Nikon A1 confocal microscope. Three dimensional (3D) volume scans (*z*-series) were recorded for analysis. 3D reconstructions were done using Nikon elements software, and further processed in high performance 3D imaging Volocity 6.0 software for the apical, basal width and cell length measurements. The average (*x,y*) coordinates were determined by outlining the apical and basal surface in Volocity software. During each cell measurement the traces of the neighboring cells was avoided by moving the *z* series from *xz* and *xy* axis. To confirm the apical, basal width and the cell length, the measurements were finalized by observing the dimensional in 3D layout (*xyz* coordinates).

### Statistics

aCaspase 3^+^ and pHH3^+^ cells were quantified in the otic, lens and olfactory epithelium in control and BMP-inhibited embryos. In the electroporated olfactory and lens epithelium only GFP-positive regions were included in the quantification. To determine the percentage of pHH3^+^ and aCaspase3^+^ cells, the number of antigen-expressing cells was quantified and compared with the total number of cells, determined by DAPI-stained nuclei. For cell death and cell proliferation studies the graphs represent the mean±s.e.m. as percentage of the total cell number. For apical, basal width and cell length measurements the mean±s.e.m. was calculated for both the controls and Noggin treated embryos. Significance was determined by Student's *t*-test with *P*-values of *P<0.05 and ***P<0.0001 accepted as statistically significant. All statistical analyses were performed using Prism 5.
